# Recurrent Fulminant Tumefactive Demyelination With Marburg-Like Features and Atypical Presentation: Therapeutic Dilemmas and Review of Literature

**DOI:** 10.3389/fneur.2020.00536

**Published:** 2020-06-30

**Authors:** Aigli G. Vakrakou, Dimitrios Tzanetakos, Theodore Argyrakos, Georgios Koutsis, Maria-Eleptheria Evangelopoulos, Elisabeth Andreadou, Maria Anagnostouli, Marianthi Breza, John S. Tzartos, Elias Gialafos, Antonios N. Dimitrakopoulos, Georgios Velonakis, Panagiotis Toulas, Leonidas Stefanis, Constantinos Kilidireas

**Affiliations:** ^1^Demyelinating Diseases Unit, 1st Department of Neurology, School of Medicine, Aeginition Hospital, National and Kapodistrian University of Athens, Athens, Greece; ^2^Department of Pathology, Evaggelismos Hospital, Athens, Greece; ^3^Research Unit of Radiology, 2nd Department of Radiology, National and Kapodistrian University of Athens, Athens, Greece

**Keywords:** tumefactive multiple sclerosis, brain biopsy, rituximab, cyclophosphamide, marburg-variant

## Abstract

Atypical forms of demyelinating diseases with tumor-like lesions and aggressive course represent a diagnostic and therapeutic challenge for neurologists. Herein, we describe a 50-year-old woman presenting with subacute onset of left hemiparesis, memory difficulties and headache. Brain MRI revealed a tumefactive right frontal-parietal lesion with perilesional edema, mass effect and homogenous post-contrast enhancement, along with other small atypical lesions in the white-matter. Brain biopsy of cerebral lesion ruled out lymphoma or any other neoplastic process and patient placed on corticosteroids with complete clinical/radiological remission. Two years after disease initiation, there was disease exacerbation with reappearance of the tumor-like mass. The patient initially responded to high doses of corticosteroids but soon became resistant. Plasma-exchange sessions were not able to limit disease burden. Resistance to therapeutic efforts led to a second biopsy that showed perivascular demyelination, predominantly consisting of macrophages, with a small number of T and B lymphocytes, and the presence of reactive astrocytes, typical of Creutzfeldt-Peters cells. The patient received high doses of cyclophosphamide with substantial clinical/radiological response but relapsed after 7-intensive cycles. She received 4-weekly doses of rituximab with disease exacerbation and brainstem involvement. She eventually died with complicated pneumonia. We present a very rare case of recurrent tumefactive demyelinating lesions, with atypical tumor-like characteristics, with initial response to corticosteroids and cyclophosphamide, but subsequent development of drug-resistance and unexpected exacerbation upon rituximab administration. Our clinical case raises therapeutic dilemmas and points to the need for immediate and appropriate immunosuppression in difficult to treat tumefactive CNS lesions with Marburg-like features.

## Introduction

Tumefactive demyelinating lesions (TDL), characterized by the presence of large (>2 cm), tumor-like lesions in CNS with perilesional edema, mass effect and/or broken ring-enhancement on MRI imaging, require a careful differential diagnosis, mainly ruling out an underlying tumor-mimicker (1, 2). The prevalence of TDLs has been reported to be 1–2 per 1000 cases of Multiple Sclerosis (3). Nevertheless, this prevalence has not been replicated in all studies due to disease heterogenicity and to the lack of appropriate registries in all countries, with studies showing a range of prevalence between 1.4–8.2% of Multiple Sclerosis patients (4). The demographics of patients with tumefactive demyelinating lesions show a slight preference of the disease to women than in men, and seems to mostly affect patients in the third and fourth decade (4). TDL may emerge during the disease course of Multiple Sclerosis (MS) or even be its first clinical presentation (Tumefactive MS) (2, 5). Interestingly, specific disease modifying drugs (e.g., fingolimod) used in MS have been associated with the appearance of TDL lesions, especially after drug-initiation or cessation (6–8). Nevertheless, TDL could represent a unique form of isolated atypical demyelinating disease, without classical radiological MS features, presenting either as monophasic disease (Monophasic TDL) or as recurrent TDL (Recurrent TDL). Studies examining the pathology of TDL lesions have shown tissue similarities with prototypical MS lesions, albeit with the unique appearance of Creutzfeldt– Peters cells and dystrophic astrocytes (9). A rare atypical demyelinating disease, that shares radiological and pathological similarities with TDL and MS, is the Marburg variant of MS (10). Marburg's disease course is considered to be monophasic, with poor response to conventional acute treatments, leading soon to death. The hallmark of Marburg's pathology are highly destructive lesions with extensive macrophage infiltration, massive demyelination, and axonal injury with overt necrosis and cavitation (9).

## Case Presentation

**Figure 1:**
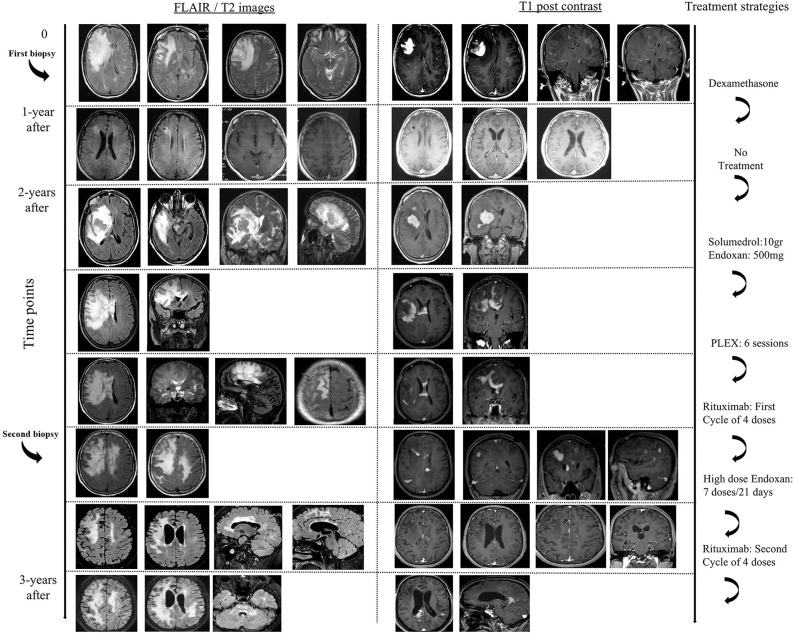
Brain MRI's in chronological order showing the effect of various treatment modalities.

We describe a 50-year-old woman, with an unremarkable past medical history, who presented with irritability, memory difficulties, severe headache and mild difficulty in gait, 1-month prior to her admission to hospital (hospitalization in another Neurology Department). Her physical examination showed left pyramidal tract signs, with a Babinski reflex on the left and motor deficit of the left upper (4+/5 MRC) and lower limb (5-/5 MRC). Magnetic resonance imaging (MRI) of the brain revealed a tumefactive right frontal-parietal lesion (T2/FLAIR hyperintense lesion), with perilesional edema, mass effect and with homogenous post-contrast enhancement, along with other small atypical lesions in the white matter (Figure 1). One of the white matter lesions in the left fronto-parietal junction displayed a ring-like appearance after gadolinium administration, whereas multiple other lesions exhibited a patchy enhancement pattern (Figure 1). Search for a primary neoplasm was negative (chest and abdominal CT scan). An extensive serological workup performed to exclude common infectious (including HIV) and autoimmune factors resulted negative. CSF analysis showed only increased IgG index (0.617), the absence of oligoclonal bands, and was negative for the detection of various common pathogens among which JC virus (PCR). Brain biopsy of cerebral lesion was performed and indicated the presence of foamy CD68-positive macrophages (macrophage rich lesion, with absence of expression of CD1a, Langerin, CD143, BRAFV600E, and S-100 markers by the macrophage histiocytes), along with dense perivascular presence of a variable numbers of T lymphocytes and a small number of B cells (images not available). No reaction with the antibody SV-40 that detects polyoma virus such as SV-40, JK, BK etc. was observed. There was no histological evidence of lymphoma or any other neoplastic process. The patient was treated with high doses of dexamethasone and levetiracetam (1,000 mg per day, initiated after brain biopsy by neurosurgeons), with gradual substantial clinical (EDSS = 0), and radiological remission (TDL lesion was not evident and only some T2-hyperintense areas persisted). A follow up brain MRI, 1 year after, also revealed resolution of the intraparenchymal mass lesion.

Two years after disease initiation, the patient experienced memory deficits, mild disorientation in time and space, gait instability and left upper extremity weakness, 15 days before her admission to our hospital. Neurological examination showed left hemiparesis. Brain MRI imaging revealed reappearance of a tumor-like mass, in the same site as the initial presentation, with a greater mass effect and strong contrast enhancement (almost homogeneous) (Figure 1). Spine MRI was normal. Evaluation of serological parameters, including AQ4 (Euroimmun, Lübeck, Germany) and MOG abs (live cell-based assay), was negative, except for the presence of serum monoclonal IgA (k) type paraprotein (11). Bone marrow examination revealed findings compatible with MGUS (less that ~5% were CD138+ plasma cells).

The patient was placed initially on dexamethasone (12 mg/days) treatment due to the mass effect of lesion and then was initiated on intravenously administered pulses of Methylprednisolone (total 10.5 gr), followed by tapering with oral methylprednisolone. The patient exhibited significant clinical improvement along with partial radiological remission of the tumefactive lesion, that showed significant reduction in contrast enhancement. We considered that our patient suffered an aggressive form of tumefactive demyelination, with Marburg -like characteristics and decided to start her on a cyclophosphamide protocol. Nevertheless, during routine laboratory evaluation, we observed an asymptomatic elevation of liver enzymes (AST, ALT). The patient was evaluated by a gastroenterologist, underwent ultrasound imaging and placed on ursodeoxycholic acid, considering that liver dysfunction was associated with high doses of corticosteroids. Due to recurrence of left hemiparesis, 3 months after the second clinical attack, our patent received 750 mg Methylprednisolone and a low dose of cyclophosphamide (500 mg), that led to further deterioration of liver enzymes and no clinical response. At that time point a magnetic resonance spectroscopy (MRS) study of the frontal lobe lesion was performed, and was consistent with a demyelinating central nervous disease {high choline (Cho)/creatine (Cr) = 6.4, high Cho / N-acetylaspartate (NAA) = 2.57, high NAA/Cr = 1.4 ratio within the center of the lesion, presence of glutamic acid, lactate, and lipids}. We decided to switch to less hepatotoxic treatments, and therefore placed our patient on PLEX (plasma exchange) sessions (*n* = 6). PLEX was unable to control the disease burden and a new brain imaging showed an expansion of the tumefactive lesion that involved more white matter areas, with more pronounced insult of the centrum semiovale, expansion to the left hemisphere, extensive invasion of the cortex and more Gd (gadolinium)-enhancing areas (Figure 1). Taking into consideration the published therapeutic effect of Rituximab on TDL, we decided to continue our therapeutic efforts with cycles of Rituximab (12–14). Our patient received 4 cycles of Rituximab (600 mg every week for 1 month) and remained clinically stable up till the third cycle. Disease exacerbation was observed following the 4th dose of Rituximab with worsening of hemiparesis and signs of intracranial hypertension (Figure 1). Interestingly, our patient relapsed while the total number of peripheral CD20 cells were 0. She received rescue therapy with mannitol and 6 gr of Methylprednisolone with modest clinical improvement.

**Figure 2:**
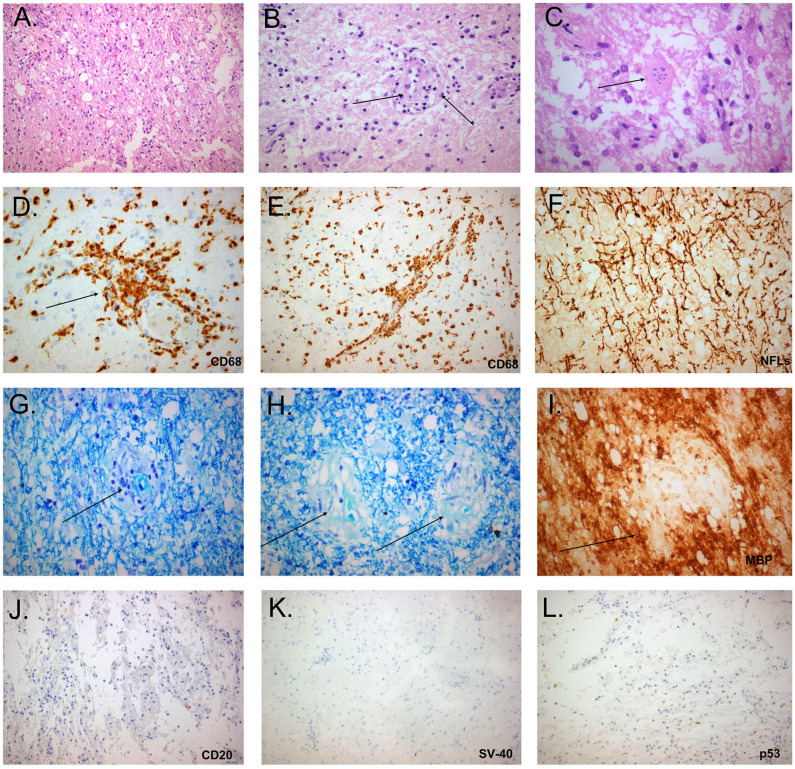
Histopathological study of the tumefactive lesion showing active demyelination (second biopsy). Immunohistochemical (IHC) findings of parenchymal inflammatory infiltrates were invariably present and mainly consisted of macrophage rich lesions **(A)**, accompanied by foamy macrophages with perivascular cuffing (**B**, black arrow) and astrocytes with granular mitosis, also known as Creutzfeldt-Peters cells (**C**, black arrow: Creutzfeldt cell with fragmented micronuclei) (hematoxylin and eosin stain). Tissue section with extensive macrophage infiltration (**D**, IHC for CD68/clone PGM1), with mainly perivascular location (**E**, black arrow). Relative axonal preservation (IHC stain for neurofilaments) **(F)**. Demyelination was observed as a loss of Luxol fast blue staining (Kluver-Barrera stain), and was most obvious in perivascular areas (**G**, black arrow, region of pallor, indicating myelin loss). Presence of granules of myelin inside the cytoplasm of macrophages, in contrast to elongated structures forming the classical myelin sheaths (**H**, black arrows, granules of myelin). Perivascular demyelination was also evident by loss of Myelin Basic Protein (MBP), in perivascular spaces (**I**, black arrow, IHC stain for MBP). Only few B cells were observed in tissue sections (**J**: IHC for CD20). Progressive multifocal leukoencephalopathy was excluded by the absence of staining with antibodies against Simian Virus-40 and p53 (**K,L**, respectively).

The extremely aggressive nature of the disease and the resistance to intense therapeutic options prompted us to reconsider the diagnosis and perform a second brain biopsy (1 month after the 4th dose of Rituximab). Histopathologic analysis revealed inflammatory demyelination particularly in areas of perivascular cuffing, predominantly consisting of CD68 macrophages (Figure 2). Parenchymal and to lesser extent perivascular infiltrates composed of small number of T lymphocytes, with relatively fewer B cells (Figure 2). Moreover, we observed the presence of reactive astrocytes with concomitant presence of nuclei with 'granular mitosis', typical of Creutzfeldt-Peters cells, frequently seen in tumefactive demyelinating lesions. Again, both lymphoma and Progressive multifocal leukoencephalopathy (PML) were excluded (Figure 2). The possibility of “ghost lymphoma” (i.e., vanishing of primary central nervous system diffuse large B cell lymphoma after steroid treatment) could not be excluded totally, but did not seem plausible, especially since it was not observed in two different biopsies of the patient (Figure 2).

Given that the patient's liver enzymes returned to normal values and the scenario of a CNS lymphoma seemed remote, we placed our patient on high doses of cyclophosphamide (1 gr/${\text{m}}_{,}^{2}$ every 21 days), for the ensuing consecutive 7 months, along with prophylactic antibiotics. Our patient responded in a dramatic fashion to cyclophosphamide infusions, with initial improvement of her clinical condition, and without manifesting progression of the disease. Her residual deficits included mild left sided disability with an EDSS of 2. Brain MRI showed a reduction in the mass effect, as well as a reduction of contrast enhancement of prior lesions (Figure 1). Nevertheless, a new relapse (3 years after the first clinical attack) occurred after the seventh dose of cyclophosphamide, with expansion of lesions to the brainstem. She was placed on Rituximab with no clinical response. She deteriorated clinically, and finally died from complicated pneumonia after a long hospitalization period (5-months) (Figure 1). Post-mortem brain tissue examination was indicative of demyelinating lesions of the CNS without evidence of a neoplastic process.

## Discussion

Fulminant demyelinating diseases represent a diagnostic challenge for clinicians and include acute disseminated encephalomyelitis, multiple sclerosis variants (Marburg variant, Tumefactive MS, Balo's concentric sclerosis) and neuromyelitis optica spectrum disorders (15). Herein we presented an extremely rare case with recurrent TDL appearance on brain imaging, with atypical tumor-like features (mass effect, homogenous enhancement, recurrence in the same anatomical site and expansion), with initial response to high doses of steroids and cyclophosmamide but subsequent resistance, with exacerbation after rituximab administration, and finally with fatal outcome. Tumefactive demyelination is considered to be not a distinct disease entity but rather a type of lesions encountered in different disease settings with Multiple Sclerosis and its variants to be the most prevalent (5). Nevertheless, TDL might occur in patients with other atypical demyelinating syndromes such as Acute Disseminated Encephalomyelitis (ADEM), AQ4 IgG seropositive or MOG-seropositive Neuromyelitis Optica Spectrum Disorders (NMOSD), as well as other neuroinflammatory disorders, such as Neurosarcoidosis or Behçet's disease (4). Of note, the spectrum of MOG antibody-associated encephalomyelitis the last years has expanded to involve cases with TDL and ADEM-like presentation, clinical presentations that could fit to our patient history (11, 16). Nevertheless, our patient was negative for both MOG and AQ4 autoantibodies. Another question regarding the differential diagnosis of our case is whether the observed demyelinating lesions could be encountered as part of a paraneoplastic syndrome or as part of an autoimmune encephalitis (17, 18). Screening for an underlying malignancy with whole body computed tomography and onconeural antibodies was negative. Particularly anti-NMDAR encephalitis has been recently associated with demyelinating events as concurrent or independent episodes (19–21). Whether the radiological and pathological differences between above disorders are sufficient to define them as separate diseases remain unclear. Till today a limited number of specific biomarkers exist to distinguish between the various demyelinating syndrome subtypes. The emergence of NMDAR, AQ4, and MOG IgG autoantibodies changed our diagnostic and treatment strategies, but more research is needed to expand our knowledge about these disease entities. An extended assessment for underlying infection, auto-immune and oncological disorders was performed in our case to rule out the various differential diagnosis, which is presented in Table 1 (15, 17, 22–26). The main differential diagnosis that remained was among tumefactive MS and the Marburg variant of MS.

**Table 1:** Differential diagnosis of pseudotumoral lesions in brain MRI (tumors excluded).

**Differential diagnosis of pseudotumoral lesions in brain MRI (tumors excluded)**	**Disease entities**
**CATEGORY**	
Multiple sclerosis	During disease course
	Upon drug initiation (fingolimod)
	Upon drug cessation (fingolimod, tysabri)
Atypical MS	Balo's concentric sclerosis
	Schilder's sclerosis (myelinoclastic diffuse sclerosis)
	Marburg varinat
	Acute hemorrhagic leukoencephalitis (AHL)
Idiopathic demyalinting syndromes	ADEM
	NMO spectrum disorders
	MOG-encephalomyelitis
	Monophasic TDL
	Recurrent TDL
Neuroinflammatory disorders	Sarcoidosis
	Behcet's disease
	IgG4 disease
	Systemic Lupus Erythematosus
	Sjogren's Syndrome
	Cerebral vasculitis (primary or secondary)
Paraneoplastic	Germ cell tumor
	Renal cell carcinoma
	Lymphoma
Infectious	HIV
	Abscess—bacterial, fungal
	Tuberculoma
	Toxoplasmosis
	Cryptococcoma
	Progressive multifocal leukoencephalopathy (PML)
	Lyme disease
	Syphilis
Genetic disorders/ leuko-vasculopathies	Genetic—retinal vasculopathy with cerebral leukoencephalopathy and systemic manifestations (RVCL-S)
	CADASIL
	Cerebral amyloid angiopathy
Inherited leukodystrophy/ leukoencephalopathy	Adult-onset leukoencephalopathy with axonal spheroids and pigmented glia (ALSP) related to CSF1R gene mutations
	X-linked adrenoleukodystrophy (ALD)
Others	Osmotic myelinolysis
	Radiation Leukoencephalopathy
	Posterior reversible encephalopathy syndrome (PRES)
	Tacrolimus
	Bevacizumab

*AHL, acute hemorrhagic leucoencephalitis; ADEM, acute disseminated encephalomyelitis; NMO, neuromyelitis optica; MOG, myelin oligodendrocyte glycoprotein; TDL, tumefactive demyelinating lesion; PML, progressive multifocal leukoencephalopathy; RVCL-S, retinal vasculopathy with cerebral leukoencephalopathy and systemic manifestations; CADASIL, cerebral autosomal dominant arteriopathy with subcortical infarcts and leukoencephalopathy; ALSP, adult-onset leukoencephalopathy with axonal spheroids and pigmented glia; CSF1R, colony stimulating factor 1 receptor; ALD, adrenoleukodystrophy; PRES, posterior reversible encephalopathy syndrome*.

An atypical and fulminant form of MS is the Marburg variant of disease. It was firstly described in 1906, and the classical form of the disease is characterized by a monophasic, highly aggressive course with rapid disease progression leading to death within weeks to months (27). The major histomorphological features of the disease involve intense macrophage infiltration, widespread demyelination (not only restricted to the perivascular areas), with further evidence of necrosis and cavitation, hypertrophic astrocytes and severe axonal injury (28). The fatal outcome of Marburg is mainly attributed to brainstem involvement and to the highly intense inflammatory process that involves tumefactive lesions with mass effect, usually not responding to acute treatments (high doses of corticosteroids). The role of plasma exchange is controversial, with past reports showing improvement in some patients; however, recent data support the inefficiency or even worsening of the disease (29, 30). Regarding the highly inflammatory nature of the disease, more aggressive immunosuppression is warranted, and treatment decisions are based on prior case reports. There are some reports showing encouraging results with the use of high dose cyclophosphamide, others with mitoxantrone and a more recently published case with alemtuzumab (31–33). Nevertheless, previously reported cases were unsuccessfully treated with cyclophosphamide with death within weeks to months after treatment (34, 35). A possible explanation for the heterogeneity in cyclophosphamide response could be the different treatment protocols (dose, frequency, time to treatment) applied to patients.

Tumefactive MS is one of the rare variants of MS. There are no therapeutic guidelines, but acute treatment involves high doses of corticosteroids and if needed plasma exchange therapy (25). Long term therapy, based on case reports, involves disease-modifying treatments classically used in typical MS (36, 37). Recent evidence supports that fingolimod and natalizumab should be avoided in MS patient with tumefactive lesions due to TDL exacerbation (25). Aggressive cases have been treated with Rituximab and/or cyclophosphamide (14). Pathological features are somewhat similar to those seen in prototypical MS, but with prominent Creutzfeldt– Peters cells and dystrophic astrocytes (9).

Our patient presented both features of Marburg variant of MS (aggressive and finally fatal disease outcome, diffuse infiltrative lesions on MRI unlike typical MS lesions) and tumefactive MS (typical histopathology, recurrent TDL appearance). Nevertheless, there are atypical features for both diagnoses. In particular, regarding the diagnosis of Marburg, atypical features include the recurrent nature of disease, and the absence of overt necrosis, as well as of axonal degeneration on biopsy. Regarding tumefactive MS, an atypical feature is the eventual resistance to all conventional therapies (corticosteroids, plex cyclophosphamide, rituximab). We consider our case unique, due to its distinct neuropathological findings and the ultimately poor response to high dose immunosuppressive treatment. The main histopathological finding (from two tissue biopsies during disease evolution and postmortem biopsy) was the perivascular demyelination and the accumulation of macrophage-rich lesions in inflamed tissue specimens. B and T cells were present to a lesser extent. Interestingly, our patient suffered disease exacerbation twice after 4 cycles of rituximab without the presence of B cells in the periphery.

Various hypothesis can be made to delineate the post-rituximab relapses. Studies in mice in models of experimental autoimmune encephalomyelitis (EAE) and in human diseases such as Sjögren's syndrome or rheumatoid arthritis have shown that anti-CD20 treatment may not eliminate a fraction of memory antigen-experienced B cells, presumably in organs other than the blood (lymphoid tissues). In individuals with increased constitutive levels of the cytokine BAFF (tumor necrosis factor ligand superfamily member 13B), BAFF, a potent B cell stimulator, may promote a fast-clonal expansion of insufficiently depleted remaining B-cells. Nevertheless, peripheral blood immunophenotyping in our case, after rituximab treatment, did not reveal reemergence of B cells upon clinical relapse, indicating that other possible mechanisms independent of memory B cells may account for disease exarbetation. An alternative explanation could be the reactivation of autoreactive long-lived plasma cells, which are not targeted by rituximab. This observation is consistent with other reports of an increase in disease activity shortly after rituximab treatment in MS and NMO (38, 39). Finally, there is a cross-talk among specific subsets of regulatory B cells and cells of the myeloid lineage, resulting in a suppressive effect of B cells (IL-10 producing B cells) over the activation status and proinflammatory differentiation of monocytes (40, 41). This is highlighted in a recently published case report showing vast CNS infiltration of monocytes after administration of alemtuzumab in an NMO patient (42). Overall, these findings suggest that the therapeutic use of rituximab or other B-cell depleting therapies in various demyelinating diseases, including our case, requires high clinical vigilance, since post-rituximab effects could be variable, depending on the inflammatory milieu and the interaction with cells of myeloid lineages.

The largest case series of biopsy-proven tumefactive demyelination with 168 cases by Luchinetti et al., has shown that 14% of patients exhibited a monophasic course, and 70% of patients eventually developed definite multiple sclerosis, with the median time to relapse to be 4.8 years (43). Regarding the severity of the overall clinical course of TDLs, Staley A Brod et al., showed that patients with Tumefactive MS exhibit a benign disease course and are successfully treated with classical MS disease-modifying therapy (44). Till today there are no clear clinical/serological and/or radiographical biomarkers assessing the risk for disease evolution and conversion to clinically definite MS. This information is critically important because determines our further therapeutic strategies after the first TDL appearance. There is no consensus regarding the use of disease modifying therapies (DMT) to decrease the risk of a second clinical attack. Many clinicians suggest using DMTs only in patients with a higher risk of conversion or in patients fulfilling MS diagnostic criteria. Potential suggested risk factors described the literature, include age at disease onset, a particularly disabling first attack, the type of enhancement pattern, and the concomitant with TDL presence of other typical MS lesions (1). Regarding our case, during the initial clinical attack of TDL (hospitalization in another hospital), we postulate that there was insufficient evidence that time point for starting MS disease modifying therapy (DMT). OCBs were tested negative, brain MRI revealed white matter lesions not typical for classical MS lesions and there was almost complete clinical and radiological response to corticosteroids. Nevertheless, the natural history of the disease stresses the need for future studies assessing better the risk for disease recurrence (new serum or CSF biomarkers, IgG index).

Diagnosis of TDL does not usually involve brain tissue biopsy, since emerging neuroimaging data contribute to proper diagnosis. The employment of various techniques, such as magnetic resonance spectroscopy (MRS), Positron emission tomography–computed tomography (PET-CT) and 8F-fluoroethyl-L-tyrosine (FET) PET can differentiate between tumor or tumefactive demyelinating (45–48). Nevertheless, the accuracy of this discrimination is not always optimal and there are limitations and gray zones. In one of the largest cohorts of biopsy-proven TDLs, about 30% of biopsies were initially misdiagnosed (43). Close monitoring of disease evolution with neurologic examination, repeated MRI scans and exclusion of other etiologies with appropriate work-up, aid the proper diagnosis. Atypical MRI characteristics (pattern of contrast enhancement, mass effect, oedema), inconclusive results of MRS, inappropriate response to immunotherapy render brain biopsy necessary.

## Conclusions

Herein, we report a case of late-onset recurrent Tumefactive Demyelinating lesion (TDL) mimicking a brain tumor. The demyelinating nature of the lesion was confirmed by brain biopsy and post-portem tissue examination as well. In our extremely rare case of fulminant tumefactive demyelination, only high doses of cyclophosphamide were able to control disease for a limited time period (7-months), indicating that alternative high-dose immunosuppressive therapies (e.g., hematopoietic stem cell transplantation) could possibly exhibit a therapeutic benefit. Of note, plasmapheresis did not control the highly aggressive tumefactive demyelination. The lack of therapeutic response despite a strenuous immunosuppressive attempt points out the need for future research in this area. Our clinical case is of great importance, as it raises therapeutic dilemmas, and points to the need for immediate and appropriate immunosuppression in difficult to treat tumefactive CNS lesions. It also highlights the need for better assessment of risk factors favoring disease relapse after the first TDL attack, in order to better determine optimal therapeutic strategies. Moreover, it underlines the value of neuropathological analysis of tumefactive lesions, not only for exclusion of other alternative diagnosis, but also for revealing the type of tissue inflammation in a attempt to better define the appropriate treatment options.

## Data Availability Statement

The datasets generated for this study are available on request to the corresponding author.

## Ethics Statement

Written informed consent was obtained from the next of kin of the patient for the publication of any potentially identifiable images or data included in this article.

## Author's Note

This case submission met all of the requirements for publication under the approval of the ethics committee of Eginition Hospital (12360/2.12.2019).

## Author Contributions

AV wrote, edited manuscript, and designed figure. DT, GK, M-EE, EA, MA, MB, JT, EG, and AD collected clinical data and treated patients in the hospital. GV and PT analyzed MRI data. TA analyzed tissue biopsies by immunochemistry and critically revised manuscript. LS and CK contributed to drafting of the work and revising it critically for important intellectual content. All authors provided approval for publication of the content of the paper and agreed to be accountable for all aspects of the work.

## Conflict of Interest

The authors declare that the research was conducted in the absence of any commercial or financial relationships that could be construed as a potential conflict of interest.
